# Evaluation of two AI techniques for the detection of new T2/FLAIR lesions in the follow-up of multiple sclerosis patients

**DOI:** 10.3389/fneur.2025.1678073

**Published:** 2025-10-08

**Authors:** Milica Mastilović, Olivier Heinzlef, Christian Federau, Verónica Muñoz-Ramírez, Marie Blanchere, Jasmina Boban, Francois Cotton, Myriam Edjlali

**Affiliations:** ^1^Laboratoire d'imagerie Biomédicale Multimodale (BioMaps), Université Paris-Saclay CEA, CNRS, Inserm, Service Hopsitalier Frédéric Joliot, Orsay, France; ^2^Faculty of Medicine, University of Novi Sad, Novi Sad, Serbia; ^3^Department of Neurology, Poissy-Saint-Germain-en-Laye Hospital, Poissy, France; ^4^CRC SEP IDF Ouest, Poissy, Garches, France; ^5^AI Medical AG, Zollikon, Switzerland; ^6^University of Zürich, Zürich, Switzerland; ^7^Kyoto Prefectural University of Medicine, Kyoto, Japan; ^8^Pixyl Research and Development Laboratory, Grenoble, France; ^9^Oncology Institute of Vojvodina, Sremska Kamenica, Serbia; ^10^Radiology Department Centre Hospitalier Lyon-Sud, Hospices Civils de Lyon, Oullins-Pierre-Bénite, France; ^11^CREATIS, INSERM U1044, CNRS UMR 5220, UCBL1, Villeurbanne, France; ^12^Department of Radiology, APHP, Hôpitaux Raymond-Poincaré & Ambroise Paré, Paris, France

**Keywords:** artificial intelligence, deep learning, magnetic resonance imaging, multiple sclerosis, lesion evaluation

## Abstract

**Background:**

Multiple sclerosis is an inflammatory demyelinating disease of the CNS. Annual MRI exams are crucial for disease monitoring. Interpreting high T2/FLAIR lesion loads can be laborious. AI aids in lesion detection, and choosing between different solutions can be challenging.

**Aim:**

This study compares two distinct software, Pixyl.Neuro.MS^®^ and Jazz^®^, to assess their performance in T2/FLAIR lesion detection between two-time points.

**Methods:**

Retrospective analysis included follow-up MRIs from 35 MS patients. Pixyl.Neuro.MS^®^ automatically segments and classifies lesions. Jazz^®^ automates the reading process and image display. Two readers (15 and 4 years of experience) conducted radiological analysis, followed by AI-assisted readings. A number of new lesions (NL) and reading times were recorded, with ground truth (GT) established by consensus. AI-detected lesions were classified as true (TP) and false positives (FP). Statistical analysis used SPSS (*p* < 0.05).

**Results:**

Pixyl.Neuro.MS^®^ readings averaged 2 min 46 s ± 1 min 4 s while using Jazz^®^ 3 min 33 s ± 2 min 24 s. Over 50% of the population had a high lesion load (>20 lesions). Both software significantly improved NL detection (*p* < 0.01 for both), revealing them in more patients than standard readings. Standard reports found 8 NL in 2 patients, while AI-assisted readings detected at least 17 TP in 7 patients and rejected 61 FP lesions. GT detected 21 lesions in 19 patients.

**Conclusion:**

Both AI software have been found to enhance NL detection in MS patients, outperforming standard methods. These tools offer crucial advantages for accurate disease monitoring.

## 1 Introduction

Multiple sclerosis (MS) is a widespread inflammatory demyelinating disease characterized by the presence of demyelinating lesions predominantly located in the central nervous system, typically distributed across periventricular, cortical/juxtacortical, infratentorial, and spinal cord regions (1, 2). The global prevalence of MS has witnessed a notable increase from 2.3 million individuals in 2013 to 2.8 million in 2020, highlighting the significant burden this condition imposes on healthcare systems worldwide. Despite extensive research efforts, a definitive cure for MS remains elusive, with current therapeutic interventions primarily focused on mitigating the risk of relapses and disability progression (3–7).

MAGNIMS group recommends at least yearly follow-up MRI exams of MS patients, or more frequently if there is a modification in the treatment strategy with a 3–6 months MRI after the initiation of treatment (8). OFSEP working group (9) highlights the importance of gadolinium contrast agent deposition in the brain and suggests that contrast is unnecessary in every follow-up exam. Therefore, accurately detecting new or enlarging T2/FLAIR lesions on follow-up magnetic resonance imaging (MRI) scans serves as a critical marker for tracking the evolution of MS disease progression (8, 10, 11). However, evaluating MS lesions poses considerable challenges due to the inherent variability in lesion distribution and load among patients. Visual lesion detection, the traditional approach, is not only time-consuming but also lacks consistent reproducibility (12).

In response to this challenge, the field has witnessed a surge in the development of artificial intelligence (AI) solutions tailored to MRI imaging in MS patients. These AI applications encompass a spectrum of software aimed at lesion detection and segmentation in MRI images, leveraging both conventional machine learning and deep learning techniques (13–17). Some AI software tools perform a longitudinal analysis of images to detect any new lesions directly between two visits. In contrast, others prioritize automating the reading process using AI to provide assistance to radiologists. However, navigating the multitude of available radiological software options presents a significant challenge for clinicians and researchers alike (18, 19).

Radiological workflow in everyday clinical practice consists of several steps that cannot be skipped, including protocol adaptation, display of images, co-registration of images, image interpretation in addition to other relevant clinical data, and finally, the summarization of all relevant findings in a final report. Software like Jazz® and Pixyl.Neuro.MS® are examples of AI tools that radiologists can use to assist with various steps in their workflow. These two software have distinct functionalities: Jazz® software (20) does a co-registration of images, displaying them to the radiologist in a more simplified manner, enabling single-click switching between previous and current images; conversely, Pixyl.Neuro.MS® software (21) performs lesion segmentation, providing radiologists with an AI-generated lesion mask with color-coded lesions to indicate their evolution.

Hence, this study aims to address this question by comparing the performance of two distinct software platforms, each with its unique functionalities, in aiding T2/FLAIR new lesion detection during the follow-up of MS patients.

Our aim was to determine the efficacy of these software solutions in facilitating the detection and characterization of MS lesions on MRI scans, ultimately contributing to improved patient care and management strategies.

## 2 Materials and methods

This retrospective study included 35 patients with MS who were referred to our institution for second opinion and follow-up for severe disease progression or high burden of MS lesions. All patients provided their written informed consent. Ethical approval was obtained from the Institutional Review Board of CPP Ile-de-France VI (ID RCB: 2019-A03066-51).

### 2.1 MRI acquisition

All patients underwent both a prior and a follow-up MRI scan using the same Siemens 3 T Skyra scanner (Erlangen, Germany). The standard OFSEP protocol for imaging of MS patients was used, including 3D FLAIR, thin slice 3D T1, axial DWI and ADC, and if needed 3D T1 post contrast, SWI, T2, DTI. However, for this retrospective study only FLAIR sequences were used for lesion analysis with the following parameters: TR = 7,000 ms, TE = 2.9 ms, TI = 900 ms, slice thickness = 1 mm, dFOV = 36.1 × 25.6 cm, resolution = 256 × 240. All images were pseudonymized before analysis.

### 2.2 Image analysis workflow

To evaluate MS lesion detection, we implemented a structured three-step analysis:

#### 2.2.1 Standard clinical reading

The standard clinical reports used for comparison were generated in routine practice by different board-certified radiologists with varying years of experience. This heterogeneity reflects real-world clinical conditions and ensures that the study results are representative of daily reporting practice.

#### 2.2.2 Reassessment using Jazz^®^ software

MRI scans were reanalyzed using Jazz® (version 1.0.0, AI Medical, Switzerland), an AI-assisted software designed to facilitate lesion detection through automated co-registration of longitudinal FLAIR sequences. The software integrates a dedicated visualization environment tailored for multiple sclerosis follow-up.

**Figure 1:**
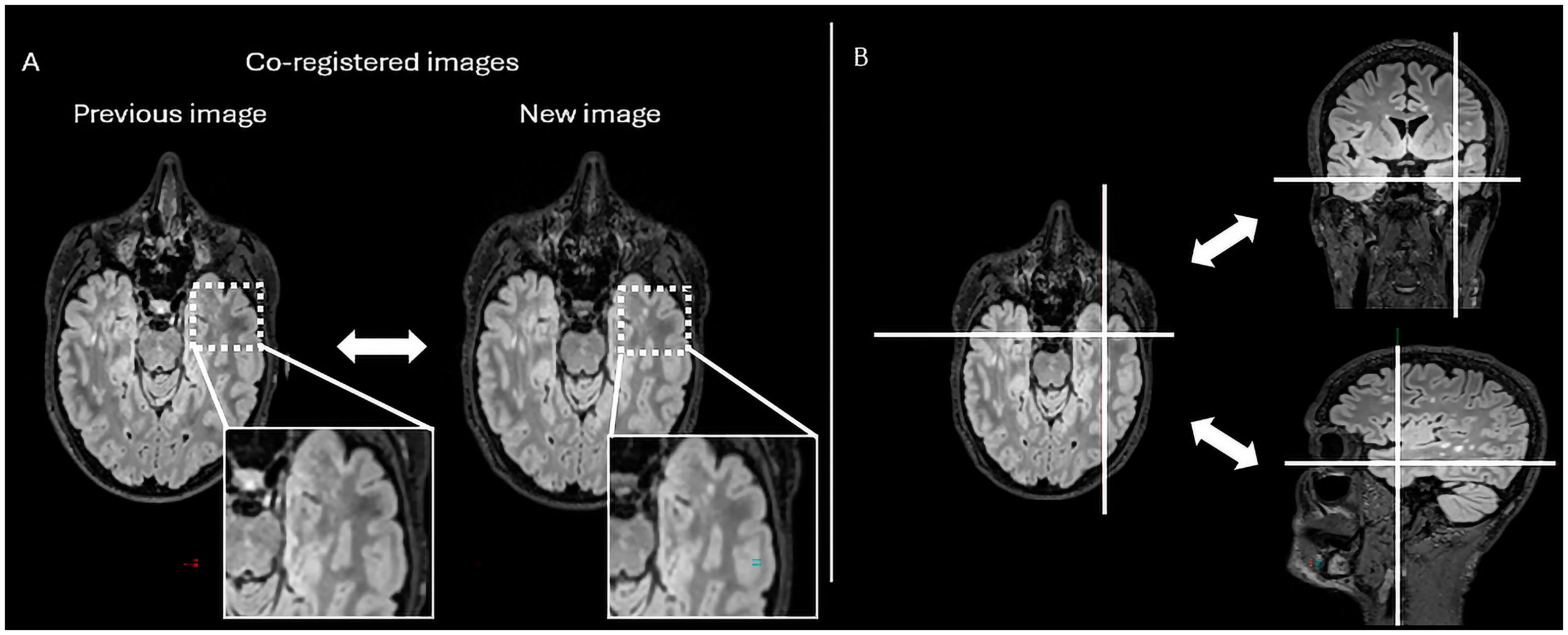
The figure represents the reading interface in Jazz^®^ software. It allows a comparison of previous and new MRI FLAIR images of a patient with MS. The reader can easily switch from previous to new image, and vice versa, just by mouse clicking or using a keyboard shortcut **(A)**, while there is as well lesion locking option **(B)** when the software automatically detects lesion’s anatomical location.

In practical terms, Jazz® enables radiologists to directly compare prior and follow-up scans at the same anatomical location, allowing instantaneous toggling between time points with a single click (mouse or keyboard shortcut). This synchronized display helps the reader identify subtle signal changes. In addition, the software provides a “lesion locking” option: once a lesion is selected, the tool automatically tracks its anatomical location across different time points, ensuring consistent assessment of potential lesion evolution (Figure 1).

For this study, two independent readers reassessed the cases with both software solutions: one senior neuroradiologist with 15 years of experience and one radiology trainee with 4 years of experience. The workflow using Jazz® software included (i) review of the paired longitudinal FLAIR images within the Jazz® interface, (ii) use of the integrated annotation tool to label new lesions, and (iii) recording of the time required for each case. At the end of the evaluation, Jazz® automatically generated a structured quantitative report summarizing the number of new lesions identified, linked to the annotated regions.

The software is designed to fit into clinical routine by allowing radiologists to quickly switch between native images and AI-assisted visualizations without altering the standard reporting process. While the primary focus in this study was on new lesion detection, Jazz® also provides quantitative lesion counts that can be exported as part of the final report.

#### 2.2.3 Reassessment using Pixyl.Neuro.MS^®^ software

Six months later, the same two readers reanalyzed the images using Pixyl.Neuro.MS® (version 1.8.7, Pixyl SAS, France) a CE-marked and FDA-cleared cloud-based medical device for quantitative brain MRI analysis. Pixyl. Neuro® is organized into several dedicated modules tailored to different clinical contexts; in this study, the MS module (Pixyl.Neuro.MS®) was used for longitudinal monitoring of multiple sclerosis patients.

The workflow is as follows: DICOM images exported from the MRI scanner are pseudonymized and uploaded to the Pixyl platform. In routine clinical practice, images can be retrieved automatically from the PACS and results are returned directly to the hospital PACS as DICOM overlays and structured reports. For this research setting, however, all analyses were launched manually, and a dedicated web interface and a secure data sharing system was used to facilitate the review of outputs.

The MS module integrates a complete processing pipeline: (i) automated image preprocessing and quality control, (ii) segmentation using a supervised deep learning algorithm based on convolutional neural networks (CNNs), and (iii) automated generation of results and reports in DICOM format.

**Figure 2:**
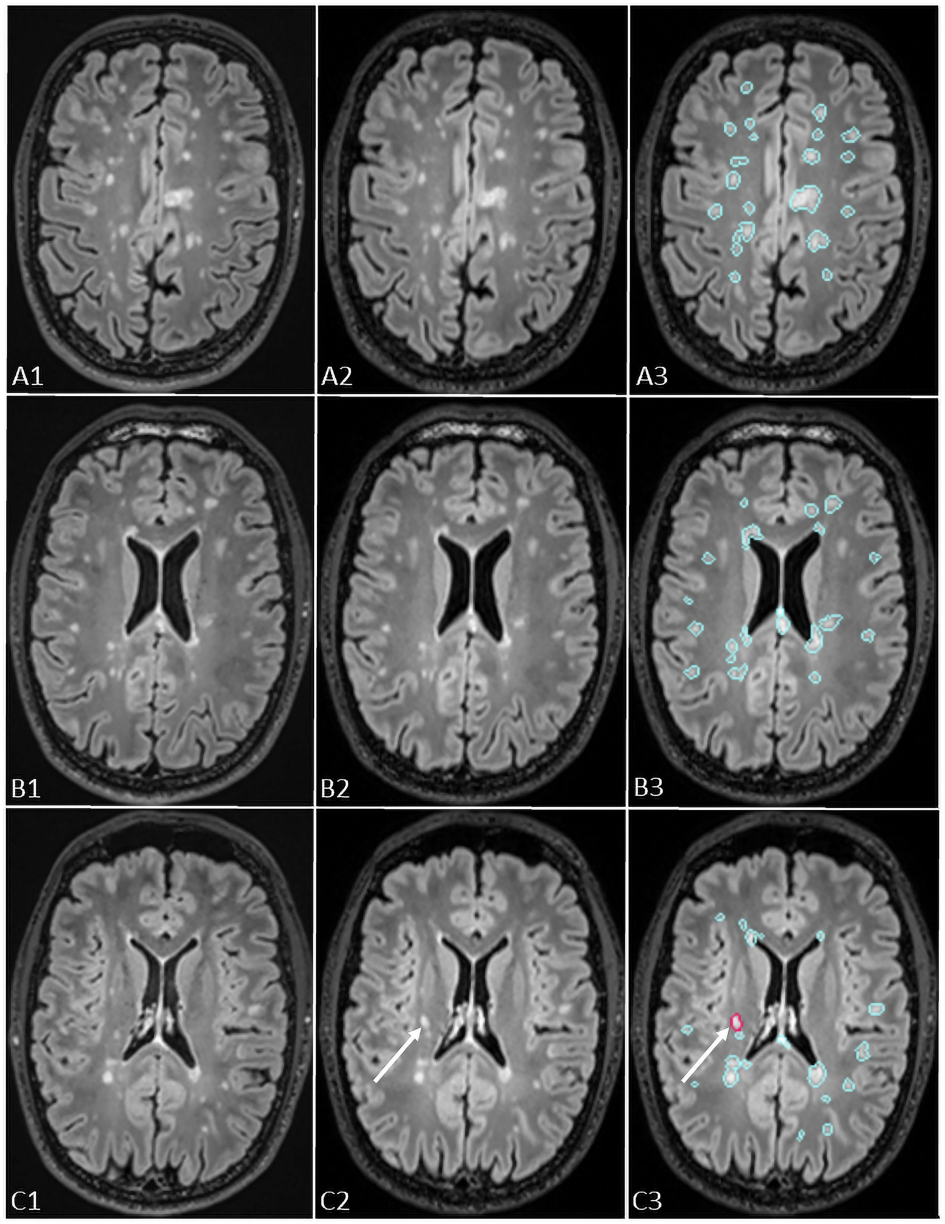
The figure shows representation of longitudinal evaluation of previous and new FLAIR images of a MS patient with high lesion load using Pixyl.Neuro.MS^®^ segmentation mask. The lesions are color-coded in the segmentation mask: blue – stable lesion, red – new lesion. The white arrows in C3 indicate a new lesion detected using Pixyl.Neuro.MS^®^, confirmed by comparison of new (C2) and previous (C1) exams.

Pixyl.Neuro.MS® processes 3D T2-FLAIR sequences independent of the MRI manufacturer. The software automatically segments white matter hyperintensity lesions and quantifies lesion load by anatomical regions. When two time points are available, its longitudinal module generates a color-coded activity map (red for new lesions, blue for stable lesions, yellow for enlarging lesions) together with a quantitative report summarizing lesion number, volume, and classification (stable, new, or growing; Figure 2).

In this study, we specifically used the longitudinal setting, and the radiologists concentrated solely on the detection of new lesions.

To prevent recognition bias, the cases were presented in a different order from the previous evaluation.

While two authors of the manuscript are affiliated with the software companies, several measures were taken to ensure an independent and unbiased validation. First, the retrospective analysis and validation were performed by authors with no ties to either company. Second, the MRI cases included in the study were not previously used for software development or training, ensuring that both AI solutions were tested on independent real-world data. Third, the AI-assisted outputs were compared against a ground truth established by experienced neuroradiologists who had no involvement in software development. Together, these safeguards minimize the risk of industry bias and strengthen the objectivity of our findings.

### 2.3 Ground truth definition

The final ground truth for lesion detection was established through a side-by-side review of prior and follow-up MRIs by the same two readers, who reached a consensus on the exact number of new T2/FLAIR lesions. The ground truth evaluation defined a new lesion as an area of abnormality measuring at least 3 mm in the axial plane (1), located in typical MS regions (periventricular, juxtacortical, infratentorial) or within non-specific white matter. Only 3D FLAIR sequences were used for lesion detection. AI software outputs (from Jazz® and Pixyl.Neuro.MS®) were incorporated into the consensus process to assess the validity of detected lesions.

### 2.4 Statistical analysis

Statistical analysis was performed using the SPSS Statistics 27 package (IBM, SPSS, NY). Descriptive statistics included mean, median, and standard deviation for quantitative variables, while qualitative values were expressed as ratios. The Wilcoxon signed-rank test was used to determine statistically significant differences between: (1) the number of patients with new lesions detected by ground truth versus standard radiological reports; (2) the number of new lesions detected using Jazz® and Pixyl.Neuro.MS® compared to standard reports. Significance was set at *α* < 0.05. Correlation between readers’ performances was assessed using Pearson’s correlation coefficient, with the following interpretation: r ≤ 0.19—very low correlation; 0.2–0.39—low correlation; 0.4–0.59—moderate correlation; 0.6–0.79—high correlation; 0.8–1—very high correlation. This structured approach ensures a clear workflow, minimizing bias while assessing the added value of AI-assisted lesion detection in MS follow-up imaging.

## 3 Results

### 3.1 Patient population

The patients’ demographic and MRI characteristics are given in Table 1. The analysis included 35 patients (mean age 48.8 ± 12.75) with multiple sclerosis with follow-up exams. Our cohort consisted of 75% women and 25% men. Lesions load varied from less than 10 lesions to more than 20 lesions, with the majority of patients exhibiting a severe lesion load of more than 20 lesions at the cerebral level (54.29%). In comparison, 28.57% had between 10 and 20 lesions, and 8.86% had less than 10 lesions.

**Table 1:** Study population characteristics.

Demographic and MRI characteristics	
Number of patients	35
Female	26
Male	9
Age, mean (SD), years	48.8 ± 12.75
Female	50.27 ± 13.28
Male	44.55 ± 12.58
EDSS, median (interquartile range)	
Baseline	4 (2.5–6)
Follow-up	4 (2.5–6)
EDSS change	0 (0–0.5)
Time between scans, days (interquartile range)	499 (234–690)
Lesion load, number (%)	
<10 lesion	6 (17.14%)
10–20 lesions	12 (34.28%)
>20 lesions	17 (48.57%)
Clinical form, number (%)	
Relapsing–remitting	21 (60%)
Primary progressive	2 (5.71%)
Secondary progressive	12 (34.29%)

### 3.2 Inter-reader reliability

A correlation analysis revealed a high and statistically positive correlation between readers when using both Pixyl.Neuro.MS® (*r* = 0.73, *p* < 0.001) and Jazz® software (*r* = 0.75, *p* < 0.001).

### 3.3 Reading time

Reading added time using Pixyl.Neuro.MS® software took on average 2 m 46 s ± 1 m 4 s for both readers, while the reading time was on average 2 min 12 s ± 51 s for reader 1, and 3 min 26 s ± 1 min 5 s for reader 2.

Reading added time using Jazz® software took, on average, 3 m 33 s ± 2 m 24 s for both readers, while the reading time was, on average, 2 m 25 s ± 1 m 6 s for reader 1, and 4 m 41 s ± 2 m 48 s for reader 2.

### 3.4 New T2/FLAIR lesions

**Figure 3:**
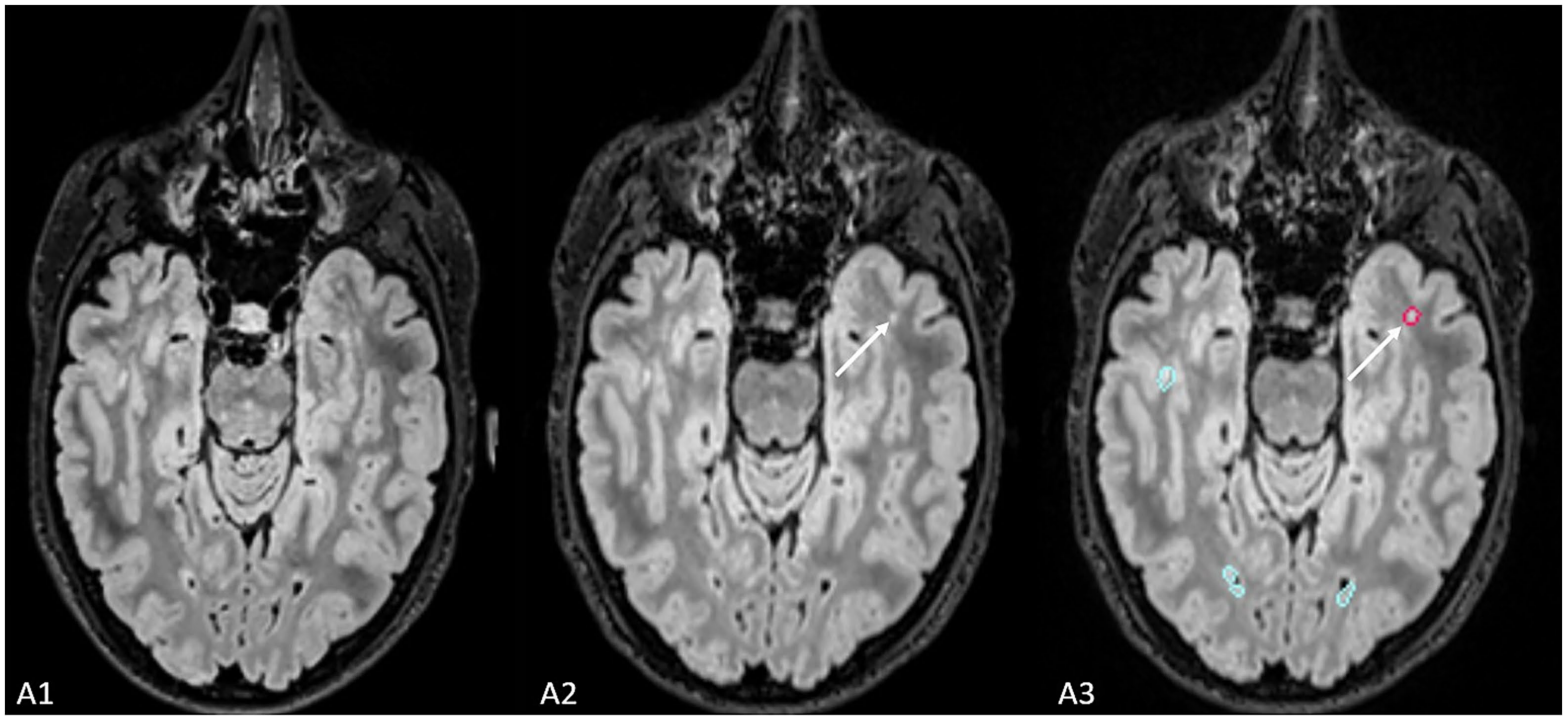
The figure shows an example of the lesion (indicated by the white arrow) missed by radiological evaluation by Jazz^®^ software, while it was detected by AI-assisted radiological report made using Pixyl.Neuro.MS^®^ software.

The ground truth reported 21 new lesions from 9 patients (Figure 3). With the use of Pixyl.Neuro.MS® software, reader 1 reported 19 true positive lesions from 9 patients, while reader 2 reported 20 true positive lesions from 8 patients. On the other hand, with the use of Jazz® software, reader 1 reported 19 true positive lesions in 8 patients, while reader 2 reported 17 true positive lesions in 7 patients. The standard report reported 8 new lesions in 2 patients.

A statistically significant difference exists between the number of patients with new lesions detected by radiological reading using Pixyl.Neuro.MS® and Jazz® software compared to the number of patients with new lesions detected by the standard radiological report (z = −2.646, *p* < 0.01 for both software).

**Figure 4:**
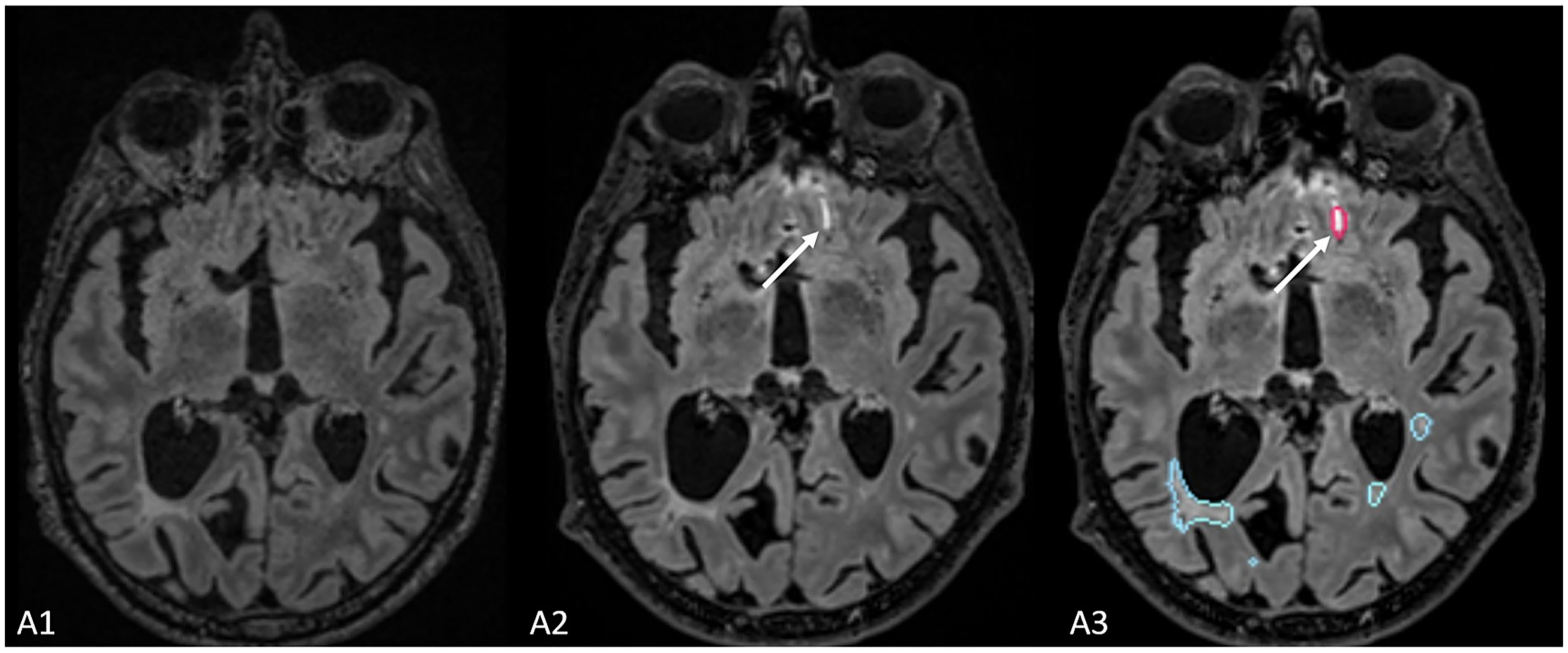
The figure represents a false positive lesion (indicated by the white arrow) which was segmented as a new lesion but actually represents an artifact located at the interface between cerebrospinal fluid and brain parenchyma.

Finally, compared to the ground truth, 61 false positive new lesions were detected by Pixyl.Neuro.MS® software alone, and radiologists did not retain these lesions (Figure 4). False positive lesions were associated with artifacts in 40% (24/61) of cases, while the remaining 60% (38/61) resulted from co-registration errors (28%) and the presence of slowly enlarging lesions (SELs; 26%).

The addition of AI support markedly improved lesion detection compared to standard clinical reports. The standard reports, produced in routine practice, showed very high specificity and PPV (100%) but low sensitivity (22.2%), reflecting the tendency to miss subtle new lesions under clinical time constraints, in a population with a large number of MS lesions per patient. When using Jazz®, both readers achieved substantially higher sensitivities (77.8–88.9%) while maintaining perfect specificity and PPV, highlighting its ability to increase lesion detection without introducing false positives. Pixyl® further increased sensitivity, reaching 100% for both readers, although Reader 2 experienced a slight decrease in specificity (96.2%) and PPV (90%), indicating a small number of false positives. When used in a fully automated mode, Pixyl® detected all true lesions (100% sensitivity and NPV) but generated a high number of false positives, leading to low specificity (26.9%) and PPV (31.2%). These results suggest that both software solutions enhance lesion detection compared to standard practice, with Jazz® favoring specificity and precision, while Pixyl® maximizes sensitivity but requires radiologist oversight to filter out false positives (Table 2).

**Table 2:** Diagnostic testing accuracy table provides sensitivity, specificity, positive predictive value (PPV) and negative predictive value (NPV) based on the patient-level.

Radiological report	Sensitivity	Specificity	PPV	NPV
Standard report	22.2%	100%	100%	78.8%
Reader 1 + Jazz	88.9%	100%	100%	96.3%
Reader 2 + Jazz	77.8%	100%	100%	92.9%
Reader 1 + Pixyl	100%	100%	100%	100%
Reader 2 + Pixyl	100%	96.2%	90%	100%
Pixyl	100%	26.9%	31.2%	100%

## 4 Discussion

In this study, AI-assisted radiological assessments significantly improved lesion detection in MS patients with a high lesion load.

Compared to standard MRI reading, both Pixyl.Neuro.MS®, which automates lesion segmentation, and Jazz®, which optimizes follow-up image display, enhanced radiologists’ ability to identify new lesions (z = −2.646, *p* = 0.01 for both software). This resulted in additional lesion detection in 20 and 22% of cases (reader 1 and reader 2, respectively), highlighting the potential of AI in challenging diagnostic scenarios. The early and accurate detection of new FLAIR lesions is one of the most important parameters for therapeutic decision-making since it helps estimate actual disease activity (22, 23). Changes in the lesion count were found to influence therapeutic decisions in various ways, potentially leading to treatment escalation in cases of breakthrough disease. However, a manual follow-up evaluation of MRI exams remains challenging for radiologists, especially in advanced stages that have a high lesion burden.

Several factors contribute to these difficulties. Initially the detection of new lesions is complicated by the multiple pre-existing lesions, especially when they are small or when their localization is in areas of tissue remodeling. Second, brain atrophy, a common feature in MS, alters brain morphology and can introduce interpretation errors. Finally, the interrater variability in neuroradiology still represents an issue which can be responsible for inconsistencies in lesion identification.

In this context, AI provides valuable assistance by improving objectivity and reproducibility in image analysis. Pixyl.Neuro.MS® and Jazz® exemplify two complementary approaches: segmentation-based versus co-registration/display-based. Pixyl.Neuro.MS® provides fully automatic quantitative outputs (lesion count, lesion volume, activity maps), offering a standardized method to track disease evolution, but at the cost of occasional false positives that require validation. Jazz®, on the other hand, provides a quantitative information but not the lesion masks, improving visual comparison between time points, thereby reducing the risk of missing subtle changes while keeping the final decision fully dependent on the radiologist.

These operational differences also imply distinct workflow consequences. In clinical practice, Pixyl.Neuro.MS® integrates with PACS and produces structured DICOM reports and overlays, which can facilitate multicenter standardization and longitudinal monitoring. Jazz® functions more as a reading accelerator, streamlining the toggling between prior and follow-up scans. The choice between the two may thus depend on whether the clinical priority is quantitative lesion tracking or rapid and confident visual assessment.

Our results align with a previous study that has explored AI’s role in MS imaging. The study of Combes et al. (24) shows the improved detection of new lesions when utilizing segmentation masks during radiological reading, whereas the study of Federau et al. (25) shows the improved detection of new lesions using AI-based software for the longitudinal MRI assessments of MS patients.

However, the impact of AI on diagnostic efficiency depends mainly on data quality and algorithm training. Conversely to the studies whose focus was on moderate lesion load cases, our study specifically investigated patients with a high lesion burden, where AI’s added value is particularly relevant. Our cohort was intentionally biased toward patients with a high lesion load, as the clinical advantage of AI-assisted detection is most pronounced when manual identification of new lesions becomes more difficult due to the sheer number of pre-existing lesions. While previous AI studies in inflammatory conditions have typically included mixed cohorts (26), the greatest detection challenges, and thus the most potential for AI benefit occur in cases with extensive lesion burden. This specificity could explain why the improvement in detection rate observed in our study was statistically significant.

One major consideration for integrating AI into neuroradiology is its effect on workflow efficiency. In our study, the average reading time with AI assistance was 2 min 46 s for Pixyl.Neuro.MS® and 3 min 33 s for Jazz®, compared to an estimated 4 min for neuroradiologists and 8 min for radiology residents in conventional reading reported in the literature (27). A study by Sima et al. (32) showed that the use of AI software resulted in statistically shorter reading times compared to standard radiological reading. Similar results were shown by Peters et al. (28) and Combes et al. (24), who demonstrated shorter reading times when using AI compared to reading times without AI. Even though we did not compare reading times without and with the use of AI, our results suggest a potential time-saving benefit, though this advantage may be offset by the need to verify AI outputs. While AI identifies more lesions, it can also produce false positive results, which need to be validated by radiologists (29). The majority of false positive results in our study were associated with co-registration errors, either only co-registration or linked to the presence of severe atrophy. Additionally, some SELs that were considered new in new exams were reclassified as SELs upon further radiological evaluation. We also encountered artifacts that were mistakenly identified as new lesions. These artifacts typically appear at the boundary where fluid meets the air and the bone, which cannot be completely suppressed in 3D FLAIR sequences (30). This aspect of AI validation may explain why the reading times might even increase in clinical practice.

Several limitations should be considered when interpreting our results. Firstly, our study was conducted on a relatively small cohort of 35 patients, with total of 21 new lesions, which limits the generalizability of the findings but nevertheless provides an important first step toward understanding how such software solutions may perform in clinical practice. Patients which were included in our study mostly had high lesion loads (>20 lesions), which prevented us from assessing AI’s performance in early-stage or low-burden cases. Secondly, MRI readings were performed in an already pre-prepared environment, which may not fully reflect everyday clinical workflow. A limitation of our study is that AI-assisted lesion detection was not applied under routine clinical conditions. Standard clinical reports were generated in daily practice, often under time constraints and other pressures, whereas the AI-assisted assessments were conducted in a controlled, experimental environment. This difference may partly contribute to the improved lesion detection observed with AI support. Future studies should investigate AI-assisted detection directly in clinical routine to better understand its practical impact. We evaluated only FLAIR sequences, whereas the integration of other sequences, such as DIR, DWI, and T1 post-contrast, could have an impact on the enhanced lesion characterization. Lastly, our study did not assess whether AI-enhanced lesion detection can be translatable to improve long-term clinical outcomes.

It should also be noted that since the time of this study, several new releases of Pixyl.Neuro.MS® and Jazz® have become available, with improvements specifically aimed at reducing false positives. Our results therefore reflect the performance of the evaluated version at the time of analysis, and may not fully capture the capabilities of more recent software iterations.

The use of AI tool which improves lesion detection opens a promising avenue in neuroradiology and aligns with the broader evolution toward augmented radiology, where AI assists rather than replaces radiologists. Several future directions should be explored. Firstly, AI needs to be validated in a clinical environment with a diverse patient cohort and radiologists of varying expertise levels. Secondly, in the study it was evaluated only FLAIR sequence using AI, but integration of other sequences, such as DIR, SWI or post contrast T1 sequences, could not only enhance lesion detection and characterization, but also improve generalizability of the findings. Thirdly, how AI can be better integrated into the clinical workflow in order to minimize validation time and maximize efficiency needs to be investigated. A hybrid AI-radiologist approach needs to be explored, mainly the fusion models between human expertise and AI, and incorporated into optimized double-reading protocols. Last but not least, a further investigation of the impact of AI-assisted radiological report on the clinical management of MS patients should be explored, especially concerning the treatment decision changes and patient outcomes, given the fact that number of new lesions and relapses in patients treated with interferon-beta are a useful tool for predicting the clinical disease activity in MS patients (31).

In conclusion, our study highlights the significant contribution of AI in detecting new lesions on follow-up MRI in MS patients with high lesion loads. Both Pixyl.Neuro.MS® and Jazz® significantly improved detection of new lesions compared to conventional readings. However, AI tools still require radiological validation to ensure that the results are accurate and also to eliminate the false positive results. Future AI integration must consider its impact on workflow efficiency and adaptation to clinical constraints.

AI represents a promising advancement in neuroradiology, but its clinical adoption requires further validation to optimize its performance and ensure meaningful contributions to patient care.

## Data Availability

The datasets presented in this article are not readily available because ethical considerations. Requests to access the datasets should be directed to myriam.edjlali@aphp.fr.
